# Transient evoked otoacustic emissions and distortion product in school children

**DOI:** 10.1016/S1808-8694(15)30595-4

**Published:** 2015-10-18

**Authors:** Rosângela Melo Vasconcelos, Lucieny Silva Martins Serra, Vânia Maria de Farias Aragão

**Affiliations:** 1 ^1^ MSc. Otolaryngologist - Hospital Universitário Presidente Dutra/ UFMA.; 2 Specialist in orofacial motion. Speech therapist -setor de audiologia da Clínica Integrada Médica Odontológica, CIMO.; 3 PhD in Pediatrics - USP-RP, Adjunct Professor of Medicine - Universidade Federal do Maranhão-UFMA. Universidade Federal Do Maranhão.

**Keywords:** audiometry, otoacustic emissions, school children, hearing loss

## Abstract

Past five years of age, the main complaint of children who are hard of hearing is that they have difficulty in learning.

**Aim:**

Compare these results to suspected hearing loss, through triage with the exams of evoked otoacoustic emissions (EOAE) transients (TEOAE) and by distortion product (DPEOAE), using data from audiometric exams; observe which of the procedures of EOAE better respond to school children triage.

**Materials and methods:**

To evaluate 451 school children, grade one students, from the public schools in São Luís. At school, otoscopic exams with the removal of wax and the TEOAE and DPEOAE exams were also carried on all school children. Audiometry and acoustic impedance were performed on the children who presented alterations at any point during the TEOAE and/or DPEOAE exams.

**Study Design:**

Transversal- Prospective.

**Results:**

18.6% had ear wax. As for the TEOAE and DPEOAE triage, no significant statistic difference was found when comparing the results of the exams which failed only in the TEOAE and DOEOAE with audiometric exam data, nonetheless, when comparing this failure data to both of these exams there was a significant difference (p<0.05).

**Conclusion:**

Both EOAE procedures responded well to the hearing triage in school children.

## INTRODUCTION

Hearing problems may be missed by parents and teachers; therefore it is important to systematize educational, preventive and hearing loss curative programs in order to mitigate and/or to avoid possible auditory sequelae that may impair the person's school performance^1^. Above five years of age, the major complaint of hard-of-hearing children is having learning difficulties, especially at school, where there are numerous distracting sounds and noises that serve to mask the message coming from the environment, making it difficult to learn educational content^2^.

Hearing assessment can be carried out by means of objective tests, such as otoacoustic emissions (OAE), immittance studies, and subjective tests such as tonal and vocal audiometry.

OAE is a low intensity sound energy which is amplified by the contraction of cochlear outer hair cells, which may be detected in the external ear canal. They were discovered in 197 by the hearing biophysics professor Dr. David T. Kemp from the University College, in London. They can be classified in: spontaneous - if recorded in the external acoustic meatus in the absence of sound stimuli; evoked - when energy is recorded in the external ear canal in response to a sound stimulus^3^.

Evoked otoacoustic emissions (EOAE) are classified in transient (TEOAE) - evoked by a brief sound stimulus, usually a broad spectrum click that encompasses a wide range of frequencies; distortion product (DPOAE) - evoked by two pure simultaneous tones (f1 and f2) that through intermodulation produce a distortion product response (2f1- f2); stimulus-frequency (SFEOAE) - evoked by a continuous and low intensity signal, these are less used clinically because their recording brings about much technical difficulties and the testing time is longer^3^, ^4^.

EOAE are recorded in most normal hearing individuals, regardless of age and gender. Its presence indicates that the cochlea is intact, and it may tell if the hearing of a given ear is within normal ranges. Screening with otoacoustic emissions present less false positives and less false negatives. It is a fast, reliable and non-invasive test; therefore making it the ideal test for screening purposes^4^, ^5^.

Middle ear immittance study - immitanciometry - offers a large number of practical applications. It provides somewhat accurate information about the health of the tympano-ossicular apparatus, enabling the differential diagnosis among purely sensorineural, conductive and mixed hearing loss^6^.

Audiometry is the method that introduces this Idea of metrics in audiology. Its goal is to establish the hearing threshold in each frequency. The electrical audiometer emits pure sounds of known and variable frequencies, usually between 125 and 8,000 hertz (Hz), at the same time, for each sound type, it produces known and variable intensities^7^.

Our study tried to assess auditory alterations in school aged children through two otoacoustic emission procedures, in order to avoid, as much as possible, false positive and false negative values, and also be able to observe which of the two procedures would be better to screen school aged-children, since we found in the literature only five papers discussing the use of otoacoustic emissions in the auditory screening of school-aged children^8^, ^9^, ^10^, ^11^, ^12^. Most of the authors report their experiences with evoked otoacoustic emissions in neonates, babies, adults, and elderly citizens. Normal hearing criteria was adopted throughout the analysis of audiometric results, although it is considered that cochlear alterations may be present before some audiometric alteration is found^13^.

## MATERIALS AND METHODS

This study was carried out after being authorized by the Ethics in Research Committee (CEP) of the University Hospital (protocol # 0795/04) and the Secretaria Municipal de Educação em São Luís (Municipal Secretariat of Education of São Luiz) - MA (Official paper # 028/2004).

We held a cross-sectional prospective and observational study in school-aged children of the first grade from 19 elementary schools of the São Luiz Public Education System, from August 1st through December 15 of 2005. We randomly selected 19 schools from the 52 public elementary schools of the São Luís urban area, and from each school we randomly selected 30 students, making up a total of 16,122 children from the 1st grade of the public elementary school system of São Luiz, data were collected at the Municipal Secretariat of Education of São Luiz.

First, we arranged with the schools’ principals to deliver a letter with authorization from the Municipal Secretariat of Education. At this point we explained them the importance of that research Project and we asked if we could use the library or a study room with the minimum possible noise level in order to carry out the hearing tests.

The Otorhinolaryngologist performed an otoscopic exam (Heine Otoscope), to check for alterations in the external ear canal and the ear drum.

Ear wax was removed for a better performance of the otoacoustic emissions test. When the children had hard or impacted ear wax, the parents or guardians were instructed to drop a wax softening solution in their ears for about seven days and later the wax was removed.

After otoscopy and recording of the findings, the children were submitted to evoked otoacoustic emissions, commonly used in the clinical setting (with an AuDX device, from Widex with printer). Standard equipment software was used in order to record the emissions, both the transient and the distortion product.

After hearing screening through otoacoustic emissions, the students who failed the exam at any point were referred to audiometric exam. For such evaluations, a sound treated booth from Vibrasom was used, and a GSI68 audiometer, calibrated in July of 2005 and an AZ7 immitanciometry device from Inter Acustics, calibrated in July of 2005.

Audiogram findings interpretation, as well as the characterization of clinical findings regarding the type of hearing loss were based on the criteria established by Russo and Santos^14^.

The values were presented in integers and relative numbers. The statistical analysis was carried out by the chi-squared test, with the Yates correction when necessary, and by the Fisher's exact test, considered significant when p < 0.05.

## RESULTS

We assessed 454 school-aged children between 6 and 11 years of age, 219 (48.0%) were females and 235 (52.0%) were males.

The otoscopic exam was carried out in 908 ears, of which 169 (18.6%) had impacted wax, and they all used wax softening ear drops for later ear washing and otoacoustic emission test, according to Table 1.

**Table 1:** Ears with wax in school aged children from the first grade of elementary public schools of São Luís-MA, 2005

Otoscopy	Right ear		Left ear		total	
	n	%	n	%	n	%
Without wax	371	81.7	368	81.1	739	81.4
With wax	83	18.3	86	18.9	169	18.6

Total	454	100	454	100	908	100

Of the 451 school aged-children submitted to otoacoustic exam - since in three of them it was not possible to remove the wax, 402 (89.0%) did not show alterations and 49 (11.0%) had some uni or bilateral failure, 232 were males with 31 (7.0%) alterations, and 219 were females, with 18 (4.0%) of alterations, there were no significant difference between the genders (p > 0.05), according to Table 2.

**Table 2:** Evoked otoacoustic emissions, according to gender, in first grade children from the elementary public school system of São Luís- MA, 2005

Gender	Otoacoustic emissions					
	pass		fail		total	
	n	%	n	%	n	%
male	201	44.5	31	7.0	232	51,5
female	201	44.5	18	4.0	219	48,5

total	402	89.0	49	11.0	451	100.0

p>0,05

When we analyzed the tests considering ear alterations, 818 (90.6%) ears passed and 84 (9.4%) ears failed transient otoacoustic exams (TEOAE); and in the ears that went through distortion product otoacoustic emissions (DPOAE), we observed 849 (94.0%) ears that passed and 53 (6.0%) ears that failed this type of emission, according to Table 3.

**Table 3:** Transient and distortion product otoacoustic emissions in first grade children from the elementary public schools of São Luís-MA, 2005.

Otoacoustic emissions	Right ear				Left ear				total			
	pass		fail		pass		fail		pass		fail	
	n	%	n	%	n	%	n	%	n	%	n	%
Transient	410	45.4	41	4.5	408	45.2	43	4.8	818	90.6	84	9.4
Distortion product	424	47.0	27	3.0	425	47.1	26	2.9	849	94.1	53	5.9

**Graph 1:**
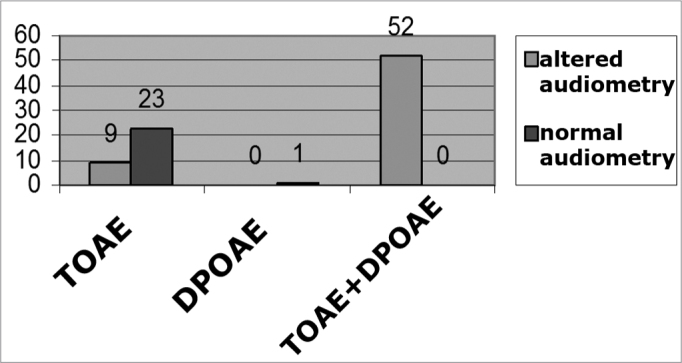
Results in absolute numbers of the failed TOAE and DPOAE isolated tests and double failure (in both tests) - TOAE+DPOAE, associated with audiometry in first grade children from the elementary public school system of São Luís-MA, 2005. - TOAE= transient otoacoustic emissions DPOAE= distortion product otoacoustic emissions

Of the 32 (3.9%) ears that failed, only the TEOAE were submitted to audiometry, and we found 9 (1.0%) with auditory alterations and 23 (2.9%) with normal audiometric exam. There was only 1 (0.1%) ear that failed DPOAE metric exams, the most frequent alteration found was the conductive type, in 56 ears (92.0%), followed by the sensorineural and the mixed types. The differences between these frequencies were significant (p < 0.05). We did not only, and when submitted to audiometry this ear presented normal values. When we compare the 52 (6.0%) exams that showed failure in both procedures (TOAE+DPOAE) they all had altered audiometry (Graph 1).

In comparing results from the audiometries of children who failed only the TOAE, with those who failed both procedures (TOAE+DPOAE), we found a statistically significant difference (p < 0.05).

In comparing results from the audiometries of children who failed only distortion product otoacoustic emission with those who failed only DPOAE, with data from the audiometries, there were no statistically significant differences (p < 0.05).

When we compared the tests that failed only TOAEs with those that failed only the DPOAEs, with audiometric tests, there was no statistically significant difference (p > 0.05).

As to the types of hearing loss found in the audio-observe any alterations between the hearing loss and the ear side (p > 0.05). Both sides presented similar frequencies in the same alterations, according to Table 4.

**Table 4:** Types of hearing loss found in audiometry of first grade children from the elementary public school system of São Luís-MA, 2005.

Types of hearing lossa	Right ear		Left ear		total	
	n	%	n	%	n	%
Mild conductive	22	36.1	23	37.8	45	73.9
Moderate conductive	06	9.8	05	8.2	11	18.0
Mild sensorineural	01	1.6	02	3.3	03	4.9
Mild mixed	00	0.0	01	1.6	01	1.6
Moderate mixed	01	1.6	00	0.0	01	1.6

Total	30	49.1	31	50.9	61	100

p < 0,05

## DISCUSSION

Evoked otoacoustic emissions are recorded from all individuals whose auditory thresholds are better than 20-30 dB HL, its presence may confirm cochlear integrity, establishing whether the otoacoustic activity of a given ear is within normal limits, and the occasional lack of otoacoustic emissions in normal ears may happen in special clinical situations, caused by anatomical alterations in the external auditory canal, or in the middle ear, or to problems associated with the equipment or the excess of environmental noise^4^.

According to the literature, transitory otoacoustic emissions (TOAE) are recorded in about 90% of the individuals with normal hearing and are more effective in the range of 1000 to 4000 Hz and with people with hearing levels between 0 and 25 dB HL^15^. International studies carried out with young individuals with normal hearing and without any otological past, found incidences of TOAE of, in an average of 98% of the population studied, coinciding with findings from a paper published by Lopes et al. with a Brazilian population submitted to similar evaluation conditions^16^. Results that coincide with the present paper found TOAE in 97.5% of school-aged children and 2.5% of ears that failed the TOAE and had their hearing within normal limits when their audiometries were compared.

Frazza^11^ studied 199 school-aged children between 6 and 10 years of age, they evaluated 358 ears and concluded that transient evoked otoacoustic emissions add the possibility of identifying sensorineural hearing losses beyond 30 dB HL, as middle ear involvement, in a rapid, objective, painless and reliable way, and can be considered a procedure of choice for hearing screening in children.

We observed that a greater impediment to acquiring otoacoustic emissions in this paper was longer response times or no response at all, depending on the noise level and the child's breathing.

We did not find significant differences in relation to gender, and this is different from the research carried out by Dell’Aringa et al.^12^ which found a higher rate of DPOAE failures in males.

In relation to distortion product otoacoustic emissions (DPOAE), they are found in almost 100% of normal individuals; however, it is possible to observe a response in people with sensorineural hearing loss up to 45dB HL^17^. We noticed an easiness and quickness in performing this type of emission, however it missed 09 individuals (0.1%), who presented alterations in their audiometric exams and failed only in the transient otoacoustic emissions.

Dell’Aringa et al.^12^, studied 93 school and pre-school aged children within the age range of 2 and 7 ears of age, through distortion product otoacoustic emissions and found a rate of 5.7% of hearing alterations, which is below the result found in the present investigation when compared to the frequency of altered otoacoustic emissions. They concluded that, otoacoustic emissions proved useful to perform screening in this age range.

We found a rate of 85 ears (9.4%) which failed the EOAE and this rate drops to 61 (6.8%) when we compare it with the results from audiometric tests. We found 2.5% of false positives in TOAEs; and 0.1% of false negatives in DPOAE.

When we do a statistical comparison between the exams that failed only the TOAE and DPOAE with the results from audiometric exams we did not find any significant difference. However, when compared to the ears that failed both types of otoacoustic emissions (TOAE+DPOAE) with data from the audiometric exams we found significant differences.

We found 18.6% of ear wax in the school aged children. When the wax was excessive and impacted, it can cause considerable hearing loss in humans. The rate of children with ear wax in the literature varies between 12 and 52%^1^, ^12^, ^18^, ^19^.

In relation to the hearing loss seen in school-aged children after audiometry, we observed a higher rate of conductive hearing loss, in agreement with the literature^1^, ^20^, ^21^.

## CONCLUSIONS

We showed that transient and distortion product otoacoustic emissions enable us to identify sensorineural and conductive hearing loss and, therefore, both one and the other can be considered as a procedure of choice for hearing screening in school-aged children.
